# TCTP in Development and Cancer

**DOI:** 10.1155/2012/105203

**Published:** 2012-05-09

**Authors:** Magdalena J. Koziol, John B. Gurdon

**Affiliations:** ^1^Department of Genetics, Yale University School of Medicine, 333 Cedar Street, New Haven, CT 06510, USA; ^2^Wellcome Trust/Cancer Research UK Gurdon Institute, University of Cambridge, Tennis Court Road, Cambridge CB2 1QN, UK

## Abstract

The translationally controlled tumor protein (TCTP) is highly conserved among animal species. It is widely expressed in many different tissues. It is involved in regulating many fundamental processes, such as cell proliferation and growth, apoptosis, pluripotency, and the cell cycle. Hence, it is not surprising that it is essential for normal development and, if misregulated, can lead to cancer. Provided herein is an overview of the diverse functions of TCTP, with a focus on development. Furthermore, we discuss possible ways by which TCTP misregulation or mutation could result in cancer.

## 1. Introduction

TCTP was first identified in tumor cells. Since its mRNA has all sequence and structural characteristics of translationally controlled mRNAs, it was named “Translationally Controlled Tumor Protein” [1, 2]. It is also known under many different names, such as histamine releasing factor (HRF), tumor protein translationally controlled (Tpt1), p23, and fortilin. The protein is highly conserved across different species [3], is ubiquitously expressed, but the level of the mRNA varies depending on the cell type [4, 5] and developmental stage [6]. A wide range of extracellular stimuli can rapidly regulate its mRNA level. Examples range from cytokines to calcium levels [7, 8]. Translational regulation of the mRNA adds another layer of TCTP level diversity [9].

TCTP expression seems to be highly regulated at many levels by many distinct mechanisms. It is not surprising that it is associated with an array of different biological activities, such as the cell cycle [3, 10], apoptosis [11–15], cytoskeleton [10, 16, 17], protein synthesis [18], immune response [19], development [6, 20–22], and cancer [11, 23, 24]. In recent years the protein has attracted most attention on account of its role in tumor reversion and its crucial role in development [21, 23]. In this paper we outline what is known so far about TCTP in development with the underlying molecular events and discuss how its misregulation might result in cancer.

## 2. TCTP Promotes Cell Proliferation and Growth

TCTP knockdown studies in *Drosophila* cause lethality in late first-instar larvae and result in reduced cell number, cell size, and organ size [21]. This indicates an effect on cell proliferation and growth, which is regulated mainly by the TOR pathway.

 The TOR pathway is regulated by nutrient and energy availability, as well as hypoxia. It integrates signals from many pathways, such as insulin signaling, growth factors, and amino acids. It not only regulates cell growth and proliferation, but also cell motility, cell survival, protein synthesis, and transcription. The pathway is named after the Target of Rapamycin (TOR), a serine-threonine kinase, encoded by the FRAP1 gene. In the presence of growth-promoting signals, receptors on the cell membrane are activated that lead to the activation of the serine-threonine kinase Akt and ultimately TOR. In mammals, the protein TOR is either bound to the protein Raptor (complex TOR1) or to the protein Rictor (complex TOR2). The TOR1 complex is sensitive to the bacterial product rapamycin and is involved in mRNA translation and ribosome biogenesis. The other rapamycin-insensitive complex TOR2 regulates cell survival and the cytoskeleton (reviewed in [25]). It regulates the cytoskeleton by stimulating various proteins, for example, actin fibers [26]. It also phosphorylates the serine-threonine kinase Akt, which initially leads to TOR activation [27].

TOR1 is activated by an increase in nutrient levels, growth factors, and stress [28]. These extracellular signals activate a cascade of proteins within the cell, leading to the activation of the GTPase Rheb, that ultimately activates TOR1. TOR1 then targets various downstream factors, such as the serine-threonine kinase S6K and the protein 4EBP1 (reviewed in [25]). S6K is known to phosphorylate many proteins. One major target is the S6 ribosomal protein. When nutrients are sparse, the S6 ribosomal protein is bound to the eIF3 complex, which is involved in the initiation of translation by recognizing the 5′ cap structure of mRNAs. mRNAs that contain a 5′ polypyrimidine tract, referred to as 5′ TOP, are important targets of eIF3 translational activation [29, 30]. These transcripts generally encode further ribosomal proteins and translation elongation factors. When the availability of nutrients increases, TOR is activated, causing an increase of S6K. Ultimately, S6 becomes phosphorylated, which causes the eIF3 complex to be released resulting in the activation of translation [31]. Various mRNAs become translated, in particular the mRNAs with a 5′ TOP region that encode proteins involved in translation. This subsequently leads to the production proteins required for translation, overall leading to the amplification of translation.

The other TOR1 target, 4EBP1, is a translation repressor. 4EBP1 binds to the eukaryotic initiation factor 4E (eIF4E), which recruits 40S ribosomal subunits to the 5′ end of mRNAs to initiate translation. Interaction of 4EBP1 and eIF4E results in the inhibition of translation. Upon TOR1 activation, 4EBP1 is phosphorylated, resulting in the dissociation from eIF4E, allowing eIF4E to initiate translation [32].

**Figure 1:**
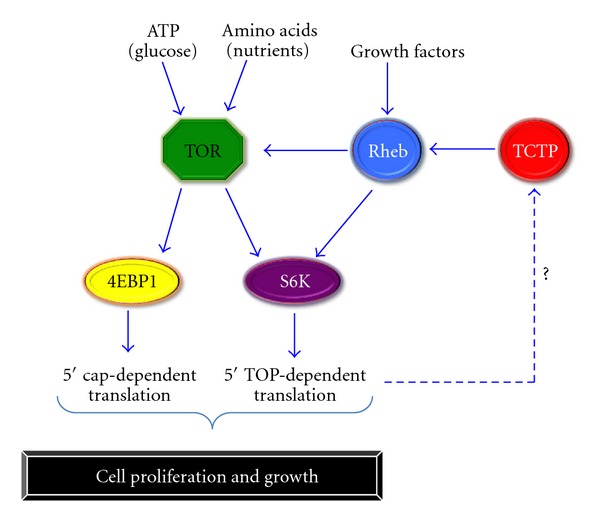
TCTP can activate the TOR pathway and promote cell proliferation and growth.

The entire TOR1 cascade and the increased protein synthesis required the activation of Rheb. Studies in *Drosophila* showed that mutant Rheb resulted in smaller cell sizes and numbers, as observed in the absence of TCTP. It was then determined that TCTP associates with Rhed. This is likely to be conserved between species, as human TCTP was able to rescue *Drosophila* TCTP mutants [21]. This observation directly links TCTP with the TOR pathway, explaining its effect on cell proliferation and growth. In the absence of TCTP, Rhed is no longer active, leading to a decrease in TOR1 activity and ultimately a decrease in protein synthesis in response to external growth-stimulating stimuli. It is known that TCTP responds to many external stimuli. This suggests that the interaction of TCTP with the TOR1 complex might be the reason for the TCTP responsiveness to many external signals [7, 8]. It would be interesting to investigate the connection of TCTP with the TOR2 pathway. Since cells are smaller in TCTP mutants, it is likely that the TOR2 pathway that also regulates the cytoskeleton is involved. To test if TCTP has an effect on the TOR2 pathway, one could analyze the effect of TCTP in the presence of rapamycin. Since rapamycin inhibits the TOR1 activity, it is possible to investigate if the absence of TCTP still has a function on the cell size and growth. If this is the case, it would be interesting to analyze the level of the major TOR2 components in the absence of TCTP. Further studies, for example, with mutants in a TCTP depleted background could help to elucidate if and where in the TOR2 pathway TCTP could act (Figure 1).

As described above, TOR1 activates the S6K kinase, which activates S6 and leads to translation of mRNAs, in particular of mRNAs that contain the 5′ TOP tract [29, 30]. TCTP mRNA itself contains the 5′-TOP domain [9]. This suggests that TOR1 might activate TCTP translation, via S6K and S6. Since TCTP activates TOR1 by binding to Rheb, the activator of TOR1, it is possible that TCTP not only activates TOR1, but also provides a positive feedback mechanism. Mutating the 5′ TOP domain of TCTP might help to determine if TCTP is actually activated by this route. If this is the case, overexpression of S6K should increase TCTP protein levels, which will in turn promote even more TOR1 activity. S6K could act as a major regulator of TOR1, since it also inactivates the repressor TOR1 by phosphorylation, suggesting a positive feedback mechanism [33]. TCTP could also act in this way, providing a positive feedback mechanism to upregulate TOR. When TCTP is high, it activates TOR1, which in turn leads to the phospporylation of S6 by S6K and increased translation. This might again lead to an increased TCTP protein level that increases TOR1 activation.

Even though abnormal cell proliferation and growth can be explained by TCTP interaction with the TOR pathway, this does not fully explain why development is ultimately arrested. This suggests that TCTP has also a major function that lies outside of the TOR pathway.

## 3. TCTP Inhibits Apoptosis

 In the early development of mice, TCTP mRNA and protein levels are significantly increased from embryonic day E3 to E5, when they reach a maximum level. Selective depletion of TCTP in the uterus at E3 resulted in reduced numbers of implanted embryos compared to wild-type embryos [34]. In knockout mice, heterozygous mutants of TCTP had no obvious developmental effects, but homozygous mutants were lethal between E9.5 and E10.5 [22]. Severe abnormalities became most prominent at E5.5, which is when TCTP level is normally at its highest. Not only did the mice embryos appear smaller, but the epiblast that eventually develops into the fetus also contained a significantly lower cell number. The reason for this was determined to be a misregulation of apoptosis [22].

 Apoptosis is a crucial part of the life of a multicellular organism. It is a highly regulated process, resulting in programmed cell death. Insufficient apoptosis may result in accumulation of mutations and uncontrolled cell proliferation, such as in cancer. Apoptosis can be induced by extracellular or intracellular signals and involves the activation of various regulatory proteins that activate the apoptotic pathway. This process is highly regulated, so that apoptosis is not induced unnecessarily, and can even be stopped if the need for apoptosis is no longer required. The intracellular apoptotic pathway is mainly regulated with the help of mitochondria, which supplies the cell with energy. A change in the permeability of the mitochondrial membrane can cause apoptotic proteins to leak into the cell. Pores called mitochondrial outer membrane permeabilization pores (MACs) regulate the permeability of the mitochondrial membrane to apoptotic proteins. Proteins belonging to the Bcl-2 protein family can regulate these MACs [35]. The protein Bax, when activated, dimerizes within the mitochondrial membrane. This dimerization promotes MAC pore formation, causing apoptotic proteins to enter the cell. In contrast, the proteins Bcl-2 and Mcl-1 inhibit MAC formation, preventing the influx of apoptotic proteins into the cell (reviewed in [36]). Apoptotic proteins that can be released via MACs into the cell are generally called small mitochondria-derived activator of caspases (SMACs). These can bind to inhibitors of apoptosis proteins (IAPs) within the cell. IAPs are usually bound to cysteine proteases that are referred to as caspases [37]. These caspases are enzymes that can degrade intracellular proteins, which ultimately cause the degradation of the entire cell. Often, these caspases need to be proteolytically cleaved in order to become active. In addition to SMACs, MAC pores also release the protein cytochrome c. Cytochrome c can then form a complex called apoptosome, by binding to ATP, the apoptotic protease activating factor1 (Apaf1) and procaspase-9. This results in the proteolytic cleavage of pro-caspase 9 into the enzymatically active form caspase 9, overall activating cellular degradation [38].

In a normal cell, the mitochondrial membrane is not permeable to SMACs. As a result, no cytochrome c is in the cell to activate caspase 9. Another class of inhibitor, the IAP proteins, are bound to caspases. Upon an apoptosis-inducing signal, the mitochondrial membrane is permeabilized, releasing SMACs and cytochrome c into the cell. SMACs bind IAPs, which release caspases, and cytochrome c converts caspase 9 to its active form (reviewed in [36]). This results in intracellular digestion and cell death. The necessity for different factors to be exported by the mitochondria shows the high level of regulation. This is not surprising, as a malfunction of the system would be detrimental to the cell.

TCTP also seems to play an important role in controlling the potentially suicidal pathway. It was found to inhibit the proapoptotic protein Bax that promotes MAC pore formation by dimerizing in the mitochondrial membrane. TCTP inserts itself into the mitochondrial membrane, preventing Bax from dimerizing [22]. This prevents MAC pore formation and inhibits any flux of apoptosis promoting factors into the cell [15]. Another study showed that TCTP also binds to Mcl-1. As discussed above, Mcl-1 inhibits MAC formation. Since binding of TCTP was found to stabilize Mcl-1, TCTP increases the block on MAC formation and ultimately prevents apoptosis [13, 14].

 It remains to be investigated what happens to TCTP when apoptosis is initiated. It is possible that the TCTP protein is actively degraded or isolated from the system, or that the TCTP mRNA level decreases. In both cases, it is likely that a factor is required for TCTP inactivation. Pull-down studies and promoter analysis when apoptosis is induced could help to find important regulators of TCTP.

## 4. TCTP in Pluripotency and Nuclear Reprogramming

During development cells become committed and differentiate from one cell into many distinct cell types. Embryonic stem (ES) cells are pluripotent cells derived from the inner cell mass of the blastocysts of an early embryo. In contrast to committed or differentiated cells, pluripotent cells can differentiate into any fetal or adult cell type and are capable of self-renewal and unlimited proliferation [39]. These have tremendous potential in medicine, as ES cells could be differentiated into any cell type or even tissue of the body and be used for potential cell replacement therapies.

 ES cells are characterized by a particular pattern of gene expression. For example, various genes are upregulated in ES cells and are frequently used as pluripotency markers. Oct4 seems to be an important regulator of pluripotency and differentiation [40]. It represses or activates expression of different genes, which occurs either directly by binding to promoter regions or indirectly by neutralising transcription activators [41]. Oct4, also known as Oct3, is a member of the POU transcription family [42]. These are transcription factors that bind via an octameric sequence to an AGTCAAAT consensus sequence [41]. The gene is expressed in early mammalian embryos, during gametogenesis, in ES cells [43], and occasionally in tumours [44]. After gastrulation, Oct4 becomes silent in mouse and human mammalian somatic cells [45]. In mouse oocytes, Oct4 mRNA is present as a maternal transcript [46] and it is downregulated when development proceeds [47]. It is essential, but not sufficient to maintain cells in an undifferentiated state [48]. During embryonic development, Oct4 is expressed in early blastomeres. Then, it becomes restricted to the inner cell mass, and is down regulated in the trophectoderm and primitive endoderm [47]. Oct4 is widely conserved. Homologues even exist in early amphibian development, where they also act as suppressors of cell fate commitment. Even though so far ES cells have not been derived from amphibians, the *Xenopus laevis* version of Oct4, Pou91, was able to fully support mouse ES cell self-renewal [49]. This suggests a similar function for Pou91 in pluripotency.

Pluripotency also requires other factors, for example, the leukaemia inhibitory factor (LIF). LIF is a key molecule required for self-renewal and pluripotency in mouse ES cells [50, 51], but not for monkey or human ES cells [52]. It is known to bind to the heterodimer LIF receptor—gp130 and to activate the transcription factor STAT3 by phosphorylation [53]. Interestingly, overexpression of the gene Nanog can bypass the requirement for LIF in mouse ES cells [54]. Nanog is also required for maintaining the undifferentiated state of early postimplantation embryos and ES cells [54, 55], making Nanog an important regulator of pluripotency. There are also other components required, such as bone morphogenic proteins (BMP) that activate the inhibitor of differentiation (Id), which represses differentiation [56]. Another important regulator is Sox2, which cooperatively binds the Oct4 protein and activates genes promoting pluripotency [57], but represses its inhibitors [58].

Despite obtaining the ES cells from blastocysts, ES or ES-cell-like cells can be obtained by nuclear reprogramming, a term introduced to describe the restoration of the embryonic pattern of gene expression [59]. Nuclear reprogramming was first demonstrated in nuclear transfer experiments. *Xenopus laevis* nuclei of differentiated cells were transplanted into enucleated frog eggs. This gave rise to normal fertile adult frogs, illustrating that differentiated cells can become reprogrammed and give rise to an entire new organism [60, 61]. Another way to reprogram nuclei was achieved when cells were fused to each other [62, 63]. Cell fusions with ES cells rejuvenated somatic cells that could differentiate into many different cell types. In these hybrids the silent gene Oct4 was reactivated [64]. Fusion experiments with an increased expression of the pluripotency gene Nanog increased nuclear reprogramming efficiency by 200-fold [65]. Nowadays, the most common way somatic cells are reprogrammed to an embryonic-like pattern of gene expression is by overexpressing different factors, such as Oct4, Sox2, c-Myc, and Klf4 under ES cell culture conditions [66]. Surprisingly, Nanog was not required, even though it seemed to promote nuclear reprogramming in cell fusion experiments [65]. These ES-like cells had normal ES cell morphology, a gene expression pattern typical for normal ES cells and could differentiate into all three germ layers. They were named iPS cells, induced pluripotent stem cells [66]. Even though the generation of iPS cells is a very convenient way to generate ES cells, this approach does not reveal the mechanism underlying nuclear reprogramming. Also, it does not identify novel factors that are involved in this process.

 To better understand the process of nuclear reprogramming, nuclear transfer experiments of somatic cells into Xenopus oocytes were carried out. It was found that even human or mouse nuclei could be reprogrammed by frog oocytes and induce an ES cell or ES cell-like pattern of gene expression [67]. For example, genes such as Oct4, Nanog, and Sox2 became transcriptionally active upon nuclear transfer [67]. Using this system, novel molecules were isolated that interact with the promoter region of Oct4. One of these molecules was TCTP. Further functional assays revealed that it in fact TCTP changed the transcriptional level of Oct4 and even Nanog in human nuclei, genes essential for successful nuclear reprogramming [68]. A similar effect of TCTP was found in bovine oocytes, suggesting a conserved function of TCTP in activating pluripotency [69]. TCTP knockout mice have an abnormal number of cells in the epiblast [22]. The epiblast is formed from the inner cell mass of the blastocyst, from which ES cells can be obtained. Since TCTP activates the pluripotency genes Oct4 and Nanog, it is possible that, in the TCTP knockout mice, the epiblast does not develop normally due to misregulation of pluripotency genes such as Oct4 and Nanog.

It would be interesting to determine if TCTP activates also other pluripotency genes such as Sox2 and Klf4. TCTP might promote pluripotency in two different ways, namely, by (1) activating pluripotency genes and (2) inhibiting somatic gene expression. Genomewide studies in the absence of TCTP could help to determine what other genes TCTP regulates. Another important question is whether TCTP is sufficient for nuclear reprogramming and if its overexpression in somatic cells could replace the four reprogramming factors used to make iPS cells. Even if it does not replace these four factors, it could increase the generation of iPS cells, a currently very inefficient process.

Nuclear actin polymerization has been reported to be required for Oct4 activation in *Xenopus laevis* oocytes [70]. Since TCTP has been found to contain an actin-binding site [17], it is possible that it might interfere with pluripotency gene regulation by interfering with actin. Testing actin polymerization in the absence and presence of TCTP, as well as the effect on Oct4, would help to understand any possible interactions required to induce pluripotency. These experiments could also be analyzed Genomewide, which will greatly help to elucidate the underlying network required to establish pluripotency. Using TCTP as bait to pull down interaction partners together with Genomewide Chromatin Immunoprecipitation analysis of TCTP and its interaction partners will also contribute towards understanding how pluripotency is established.

Another protein that has been found to interact with TCTP in Xenopus oocytes is nucleoplasmin Npm1 [71]. Similar to TCTP knockout mice, mice deficient in Npm1 are embryonic lethal and have smaller embryo sizes [72]. Npm1 is a very abundant protein. In fertilized *Xenopus* eggs, it is involved in the decondensation and hence transcriptional activation of the paternal genome provided after normal fertilization by the sperm (reviewed in [73]). It is possible that TCTP not only activates pluripotency genes, but also that it has a role in paternal gene activation by interacting with Npm1. Disturbing the interaction of TCTP and Npm1 could show if TCTP is also involved in this process. But it is possible that pluripotency and paternal and maternal genome activation is actually not that different. After all, when the genome becomes transcriptionally active, it is set as such, so that it can proliferate and differentiate into an entire organism. Hence, zygotic genome activation could be regarded as nuclear reprogramming that occurs naturally in nature, without the need of nuclear transplantation, cell fusion experiments, or overexpression of a few transcription factors.

## 5. Cell Cycle Regulation of TCTP

The cell cycle describes the stages a cell has to go through to divide and duplicate its genome. In eukaryotes, the cell cycle is divided into four phases: (1) the G_1_ phase, in which the cell grows and makes sure it is prepared for DNA replication, (2) the S or synthesis phase, where the DNA is duplicated, the (3) G_2_ phase, in which the cell ensures it is ready for mitosis, and (4) the M phase, in which cell growth stops and the cell divides its DNA and other cellular components giving rise to two cells. There is also an additional phase, which is not part of the cell cycle, G_0_, in which the cell has exited the cell cycle and has stopped dividing [74]. Since the cell cycle is crucial for the survival of the cell and generation of a multicellular organism, the process is highly controlled. There are many proteins that control each phase and that detect and repair genetic damage, as well as avoiding the propagation of mutations [74]. Any misregulation might result in uncontrolled cell proliferation and ultimately cancer. The key enzymes regulating the progression from one phase into the next are called cyclins and cyclin-dependent kinases. There are also many other proteins, such as the serine-threonine protein kinase polo-like kinase 1 (PLK1) and the protein checkpoint with forkhead and ring finger domains (CHFR).

The protein CHFR is an E3 ubiquitin ligase that can detect microtubule abnormalities. It delays the G_2_ to mitosis transition when it is exposed to altered microtubules. Microtubules are part of the cytoskeleton and act in mitosis and move the duplicated genomes into the forming daughter cells. CHFR is usually present in an inactive form, unable to carry out ubiquitination. When microtubules are damaged, CHFR becomes activated [75]. CHFR then ubiquitinates PLK1 that results in PLK1 degradation [76]. The kinase PLK1 is required in the late G_2_ and early mitotic phases. It regulates spindle assembly and centrosome maturation, which is a microtubule-organizing center. PLK1 phosphorylates and activates Cdc25C, which dephosphates and activates the cyclins required for mitosis, the cyclinB/cdc2 complex [77, 78]. Any loss of PLK1 can induce a block in cell cycle progression and lead to apoptosis. PLK1 overexpression is frequently observed in connection with centrosome abnormalities, improper segregation of chromosomes and tumor cells.

Although the TOR pathway might be indirectly involved in cell cycle regulation by responding to growth factors and energy levels and driving cell proliferation, TCTP seems to be involved more directly in the cell cycle. For example, TCTP expression is upregulated upon entry into the cell cycle, but when overexpressed, cell cycle progression is delayed [10]. TCTP also has a tubulin-binding site that allows it to bind to microtubules in a cell-cycle-dependent way. As a result, it is recruited to the mitotic spindle during metaphase, but is released at the M/G_1_ transition [10]. Furthermore, TCTP interacts with CHFR that interacts with microtubules [79]. Upon depolymerization of the microtubules, CHFR and TCTP interaction is diminished. It has been suggested that this might provide a mechanism by which CHFR senses microtubule abnormalities that results in CHFR activation, PLK1 degradation, and ultimately cell cycle arrest [79]. It would be interesting to determine if CHFR can bind with the same affinity to microtubules in the absence of TCTP, or if it is no longer sensitive to microtubule abnormalities in the absence of TCTP, confirming the proposed model. In addition to binding to CHFR, TCTP can be phosphorylated by the substrate of CHFR, PLK1 [80]. This presumably leads to a decrease in the affinity of TCTP for microtubules or CHFR. When PLK1 phosphorylation sites on TCTP are blocked, a dramatic increase in multinucleated cells is observed suggesting that the completion of mitosis is inhibited [81]. This suggests that TCTP is crucial in cell cycle regulation and that its phosphorylation by PLK1 is required for accurate exit from mitosis. In the TCTP mutants that cannot become phosphorylated, an increase in apoptosis is also observed [81]. Bearing in mind that TCTP is involved in apoptosis, it is possible that PLK1 acts *via *TCTP to inhibit apoptosis. TCTP phosphorylation by PLK1 causes cell cycle progression. It is possible that this modified TCTP might have inhibitory effects on the apoptotic pathway. In this way, TCTP could make sure that when cell cycle progresses no apoptosis is induced. In contrast, if it is not modified by PLK1 during mitosis, it might induce apoptosis via the different routes described above. It would be revealing to investigate the role of the modified TCTP protein in apoptosis.

## 6. TCTP in Cancer

**Figure 2:**
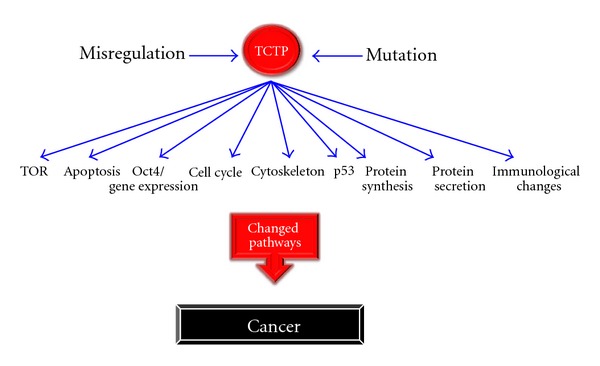
Pathways in which TCTP misregulation or mutations could cause cancer.

TCTP has been associated with tumorigenesis and cancer since its discovery in tumor cells [1, 2]. It was not until tumor reversion screens that TCTP got attention as a key player in cancer (Figure 2) [11, 23]. Tumor reversion is a process by which some cancer cells lose their malignant phenotype. Studying this process might help to understand how cancer can be inhibited and ultimately lead to a cure. To understand this process on a molecular level, tumor cells were grown in the presence of the H1 parvovirus [23]. This virus preferentially kills tumor cells, which in turn allows for selection of cells that revert back to a normal, nonmalignant phenotype [82, 83]. To identify which genes are most likely to be involved in this process, the level of gene expression was compared between malignant and reverted state. The TCTP gene expression level showed the largest difference between malignant and reverted state. A high level associates with tumorigenesis and a low level with normal cell growth (124 times higher TCTP level in tumor cells versus revertants). This was confirmed in several different tumor cell lines, suggesting that it is a universal gene that is implicated in tumor reversal [23]. Furthermore, knockdown experiments of TCTP in various malignant cell lines increased tumor reversal by approximately 30% [11].

The p53 protein is one of the most famous tumor suppressors and is often referred to as the “guardian” of cancer. It is a transcription factor and regulates the transcription of various genes. It can activate the transcription of DNA repair genes when the DNA is damaged, through genes involved in cell cycle and initiate apoptosis by regulating genes such as Bax and Bcl-2 [84]. In response to stress such as DNA damage, it either induces repair genes to repair the damage, cell cycle arrest to prevent the replication of damaged DNA, or induces apoptosis to eliminate potentially malignant cells. Various signals are responsible for whether p53 induces repair, cell cycle arrest, or apoptosis (reviewed in [85]).

To better understand how TCTP levels control cancer, the interaction between TCTP and p53 has been studied in more detail. It was found that TCTP overexpression can lead to p53 degradation. This was accompanied by the observation that p53 was no longer able to induce apoptosis [24]. This suggested that TCTP is an important regulator in the p53 pathway and also links p53 with apoptosis.

MDM2 is a transcriptional target of p53. When overexpressed, MDM2 ubiquitinates and degrades p53, providing a negative feedback mechanism. TCTP was found to inhibit MDM2 autoubiquitination and to promote MDM2-mediated ubiquitination of p53, which ultimately leads to p53 degradation [86]. In addition, p53 was found to downregulate TCTP levels [23] and to promote TCTP exosome secretion [87, 88]. This shows that p53 and TCTP antagonize each other. Similar evidence comes from a different observation. The dsRNA-dependent protein kinase (PKR) increases p53 transcriptional function [89]. Mice depleted of PKR had altered TCTP protein levels. Further analysis showed that PKR directly interacts with TCTP mRNA. This interaction is required for PKR activation [9]. Hence, the presence of PKR might sequester TCTP mRNA and remove free TCTP mRNA from the RNA pool that would otherwise be available for translation. Hence, a higher level of PKR might be associated with lower TCTP protein levels. As PKR activates p53 and counteracts TCTP, it adds another layer of antagonistic control between TCTP and p53. The level of both p53 and TCTP might determine which pathway to choose, cell cycle arrest or apoptosis.

 As outlined above, TCTP misregulation has an impact on the TOR pathway, apoptosis, reprogramming and cell cycle. All of these pathways can be implicated in cancer when they do not function correctly. In the TOR pathway, overexpression or mutations enhancing TCTP activity might result in increased TOR activation, leading to enhanced cell growth and ultimately tumor formation. Similarly, alterations in TCTP level might alter the ability of TCTP to inhibit apoptosis. Any TCTP misregulation might prevent damaged cells from being eliminated by apoptosis and in this way promote the survival of cells that might result in cancerous cells. Nuclear transfer experiments have shown that TCTP induces the transcription of pluripotency genes such as Oct4 and Nanog. An increased level of TCTP in normal cells may promote the formation of pluripotent-like gene expression. This might partly reprogram quiescent differentiated cells into pluripotent like proliferating cells. If in addition mutations accumulate in these cells, elevated levels of TCTP might enhance the propagation of these mutated cells. The higher the level of pluripotency transcripts is, the greater is the cell's malignant potential [90]. This suggests that this is also true for a higher TCTP level. Ultimately, this can result in cancer. Misregulation of TCTP might also impact cell cycle progression by interfering with PLK1. PLK1 is overexpressed in a range of human tumors, and PLK1 overexpression is associated with a bad cancer prognosis [91]. Since PLK1 phosphorylates TCTP that is required for cell cycle progression from mitosis, it is possible that an overexpression of PLK1 causes faster TCTP phosphorylation and cell cycle progression. This faster cell cycle progression might result in cell cycle progression even when mitosis is not complete. The resulting daughter cells could in this way inherit not fully replicated genomes. This might result in a vast amount of mutations that might result in cancer. Alternatively, TCTP could be mutated, being irresponsive to PLK1, and have the same effect.

Finally, TCTP is also known to be involved in protein synthesis by acting as a guanine nucleotide dissociation inhibitor for the elongation factor EF1A [18]. Any changes in TCTP level could influence many genes at once and change the status of a cell substantially. Similarly, changes in TCTP will also affect the immune response and ultimately might promote cancer development [19].

## 7. Conclusion

In summary, TCTP is highly conserved and abundant. It is involved in many key biological pathways, such as the TOR pathway, apoptosis, nuclear reprogramming, and cell cycle. It is highly regulated on a transcriptional, translational and protein level. As TCTP is involved in a wide array of biological functions, it is not surprising that any changes to TCTP might result in an array of abnormal phenotypes. Furthermore, abnormal cell proliferation, growth, and survival are probably the most important characteristics of cancer, all of which are regulated by TCTP. Due to this involvement, and its presence in many other pathways, TCTP might be a crucial target for cancer therapies. Some success has already been reported in this regard [11]. Bearing in mind that TCTP is also a histamine-releasing factor, inhibitors of this pathway were tested for their ability to decrease the number of tumor cells by inhibiting TCTP. In fact, many of such inhibitors were found to kill tumors [11]. However, further studies are required to better understand the function of TCTP in the pathways described before and maybe to reveal further functions. Overall, this will greatly contribute to the understanding of basic molecular pathways and provide further target sites for cancer therapies.
